# Dermal-Type Macrophages Expressing CD209/DC-SIGN Show Inherent Resistance to Dengue Virus Growth

**DOI:** 10.1371/journal.pntd.0000311

**Published:** 2008-10-01

**Authors:** Wing-Hong Kwan, Erika Navarro-Sanchez, Hélène Dumortier, Marion Decossas, Hortense Vachon, Flavia Barreto dos Santos, Hervé W. Fridman, Félix A. Rey, Eva Harris, Philippe Despres, Christopher G. Mueller

**Affiliations:** 1 CNRS, Laboratory of Therapeutic Immunology and Chemistry, IBMC, Université Louis Pasteur, Strasbourg, France; 2 INSERM U872, Centre de Recherches Biomédicales des Cordeliers, Paris V UMRS 872, France; 3 INSERM U872, Centre de Recherches Biomédicales des Cordeliers, Paris VI UMRS 872, France; 4 Unité Interactions Moléculaires Flavivirus-Hôtes, Institut Pasteur, Paris, France; 5 Unité de Recherche de Virologie Structurale, CNRS URA 3015, Institut Pasteur, Paris, France; 6 Division of Infectious Diseases, School of Public Health, University of California, Berkeley, California, United States of America; George Washington University, United States of America

## Abstract

**Background:**

An important question in dengue pathogenesis is the identity of immune cells involved in the control of dengue virus infection at the site of the mosquito bite. There is evidence that infection of immature myeloid dendritic cells plays a crucial role in dengue pathogenesis and that the interaction of the viral envelope E glycoprotein with CD209/DC-SIGN is a key element for their productive infection. Dermal macrophages express CD209, yet little is known about their role in dengue virus infection.

**Methods and Findings:**

Here, we showed that dermal macrophages bound recombinant envelope E glycoprotein fused to green fluorescent protein. Because dermal macrophages stain for IL-10 *in situ*, we generated dermal-type macrophages from monocytes in the presence of IL-10 to study their infection by dengue virus. The macrophages were able to internalize the virus, but progeny virus production was undetectable in the infected cells. In addition, no IFN-α was produced in response to the virus. The inability of dengue virus to grow in the macrophages was attributable to accumulation of internalized virus particles into poorly-acidified phagosomes.

**Conclusions:**

Aborting infection by viral sequestration in early phagosomes would present a novel means to curb infection of enveloped virus and may constitute a prime defense system to prevent dengue virus spread shortly after the bite of the infected mosquito.

## Author Summary

Mosquito-transmitted pathogens are a major challenge to humans due to ever-increasing distribution of the vector worldwide. Dengue virus causes morbidity and mortality, and no anti-viral treatment or vaccine are currently available. The virus is injected into the skin when an infected mosquito probes for blood. Among the skin immunocytes, dendritic cells and macrophages are equipped with pathogen-sensing receptors. Our work has shown that dermal macrophages bind the dengue virus envelope protein. We demonstrate that monocyte-derived dermal macrophages are resistant to infection and present evidence that this is due to sequestration of the virus into fusion-incompetent intracellular vesicles. This identifies skin macrophages as the first innate immune cell potentially capable of protecting the human host from infection by dengue virus shortly after a mosquito bite. These findings have important implications for better understanding the early infection events of dengue virus and of other skin-penetrating pathogens.

## Introduction

Dengue is probably the most important mosquito-transmitted viral disease of humans worldwide. It is caused by dengue virus (DV), which exists as four serotypes (DV1-4) and circulates in an endemic-epidemic mode in most tropical and sub-tropical territories. Transmission of DV to humans occurs when an infected mosquito probes for blood vessels and during a blood meal, through injection of infectious saliva into the human dermis. As a member of the *Flaviviridae* family, DV infection involves virus uptake into endosomal vesicles that undergo acidification. The low pH induces structural alterations in the envelope (E) protein that lead to membrane fusion and the release of the nucleocapsid into the cytoplasm [1]. After uncoating, the RNA genome is translated to initiate virus replication. It has been proposed that non-neutralizing antibodies raised against one DV serotype may enhance infection by a heterotypic serotype [2]. This may explain why secondary infections are often associated with the more severe forms of dengue fever (hemorrhagic fever with or without shock).

Much research on DV relies on relevant human cell culture models due to the difficulty of establishing appropriate animal models. Progress has been made by showing that DV E protein recognizes the C-type lectin CD209 and its homologue L-SIGN and that expression of either of these lectins is sufficient to render cells permissive to DV grown in mosquito cells [3],[4]. Recently, the mannose receptor (MR) has also been shown to mediate DV binding and infection [5]. Dendritic cells (DC), generated from monocytes in the presence of GM-CSF and IL-4, express CD209, L-SIGN and the MR and are highly susceptible to DV infection [3],[4],[6]. These monocyte-derived DC are thought to be representative of dermal DC (dDC), yet there is increasing evidence that CD209 is not expressed by dDC but primarily by dermal macrophages (dMφ) [7]–[9]. This underscores the importance of dMφ in early infection events and raises the question of whether dMφ are permissive for productive DV infection. Studies of these cells have been hampered by the lack of suitable isolation techniques from human skin and culture methods to generate the cells from monocytic precursors.

Here, we confirmed that human dMφ express CD209 and showed that they bind DV E protein. Based on the finding that dMφ stained for intracellular IL-10, we developed a method to generate the cells from monocytes in the presence of IL-10. The monocyte-derived dMφ bound E protein and acquired DV in intracellular vesicles, but were resistant to viral replication. The inability of DV to grow in these dermal-type Mφ was attributable to accumulation of internalized virus particles into poorly-acidified phagosomes. These findings advance our understanding of the host innate resistance to DV at the early stages of infection and have implications for other pathogens recognizing CD209.

## Materials and Methods

### Dermal cell suspensions

Before blood and tissue samples were collected for the study, all healthy donors and patients gave written informed consent in agreement with the Helsinki Declaration and French legislation. A prospective IRB approval was not obtained since there was no need as specified by French law of the health protection act when employing healthy material destined for disposal or one-time biomedical research. A retrospective IRB approval was given. Fresh skin (about 50 cm^2^) was obtained from patients undergoing breast reduction surgery or abdominoplasty. The skin was trypsinized to peel off the epidermis and the remaining dermis was processed as described elsewhere [10] with the modification that only collagenase type I (1 mg/ml, Invitrogen) was used for 18 h at 37°C. The resulting cell suspension was pipetted and serially filtered through 100 µm and 70 µm cell strainers (BD Biosciences) to remove undigested tissue fragments and to obtain a homogeneous cell suspension.

### Protein sE-eGFP production

A DNA fragment containing the DV3 genomic region (Swiss-Prot accession number P27915) coding for the prM-E protein (1674 nt in total, including all of prM and the E ectodomain, ending at codon 392 of E, at the end of domain III) was amplified by PCR with forward primer 5′TTATGCATATTACTGGCCGTCGTGGCC and reverse primer 5′CTCGCCCGCAGACATGGCCTTATCGTCATCGTCGGGCCCCTTCCTGTACCA-GTTGATTTT and inserted into the plasmid pT352. This is a shuttle vector containing selection markers for yeast and *E. coli*, as well as a metallotheionein-inducible expression cassette for *Drosophila* cells. In the construct, called pT352/DV3 sE-GFP, the DV prM-E sequence is in-frame with the Drosophila BiP signal peptide, which directs the recombinant protein to the secretory pathway. *Drosophila* S2 cells (Invitrogen) were co-transfected with pT352/DV3 sE-GFP and a vector conferring resistance to blasticidine, using the effectene transfection reagent (Qiagen). The selected cells were adapted to serum-free growth medium and grown to high density before induction with CuSO_4_ at 500 µM. The supernatant was collected 10 days later and concentrated using a flow concentration system with a 10 KDa-cutoff membrane (Vivascience), and DV3 sE-GFP was purified by affinity chromatography using a Steptactin column. The eluate was concentrated and further purified by size-exclusion chromatography, using a Superdex 200 10/300 column (GE Healthcare) with 0.5 M NaCl and 50 mM Tris (pH 8.0). Purified DV3 sE was concentrated to 10 g/liter in Vivaspin ultrafiltration spin columns (Sartorius).

### DV3 sE protein binding and CD209 expression of dermal cells

Dermal cells were collected 48 h after culturing in complete medium, RPMI medium supplemented 10% fetal calf serum (FCS) and antibiotics (Invitrogen), and 3×10^5^ cells were incubated with 1, 2, 4 or 8 µg recombinant DV3 sE-eGFP fusion protein in 0.1 ml complete medium at 37°C for 30 min. The cells were then washed twice with complete medium and incubated with anti-CD14-APC, anti-CD1a-PE and anti-HLA-DR-PerCP mAb (BD Biosciences) in PBS/2% FCS for 15 min. Following 3 washes, the cells were fixed in 0.4% formaldehyde and analyzed by flow cytometry (FACS Calibur, BD Biosciences). The relative MFI for 3 donors was determined in triplicate after gating for CD1a^+^HLA-DR^+^ or CD14^+^HLA-DR^+^ cells using the following formula: (MFI (FL1) protein sE-eGFP – MFI (FL1) no protein sE-eGFP)/MFI (FL1) no protein sE-eGFP. To determine CD209 expression, 3×10^5^ cells were incubated with anti-CD209-PerCPCy5.5 (clone DCN46, BD Biosciences), anti-CD14-APC, anti-CD1a-PE and anti-HLA-DR-PerCP mAb in PBS/2% FCS for 15 min and, after washing, fixed and analyzed by flow cytometry.

### Immunofluorescence of skin sections

Formaldehyde-fixed, paraffin sections were rehydrated and antigen was retrieved in citrate buffer pH 6 at 97°C for 45 min. Biotin was blocked using the avidin-biotin blocking kit (Vector Inc.), and sections were saturated in 5% human serum at room temperature for 40 min. The following primary Abs were used: goat-anti IL-10 (1∶75 dilution, R&D Systems), mouse anti-CD209 (2 µg/ml, R&D Systems), mouse anti-CD1a (Immunotech) and mouse anti-CD14 (1∶40 dilution, Novocastra). The secondary Ab (Jackson) were: biotin-conjugated donkey anti-goat followed by streptavidin-Alexa 488 (Molecular Probes-Invitrogen) and F(ab)'_2_ rabbit anti-mouse followed by Cy3-conjugated donkey anti-rabbit. Sections were observed by confocal microscopy (LSM510 Zeiss).

### Cell culture and phenotypic analysis

Monocytes were isolated from 200 ml of adult human peripheral blood using negative-depletion beads (Dynal-Invitrogen) or by counterflow centrifugal elutriation. To obtain MDdMφ, 3×10^6^ monocytes were cultured for 5 days in 5 ml of complete medium containing 10 ng/ml M-CSF (R&D Systems), 20 ng/ml IL-10 (Immunotools) and 20 ng/ml GM-CSF (Schering-Plough) with refreshment of GM-CSF (10 ng/ml) and IL-10 (10 ng/ml) at day 3. For MDDC, 3×10^6^ monocytes were cultured for 5 days in 5 ml of complete medium containing 50 ng/ml GM-CSF and 10 ng/ml IL-4 (Schering-Plough) with readdition of cytokines at day 3. Non-adherent cells were harvested. Expression of markers was measured by FACS using specific antibodies and their corresponding isotype controls. To assay for DV3 sE protein binding, cells were pre-incubated for 10 min in complete medium in the absence or presence of 5 mM EDTA before adding 3 µg DV3 sE-eGFP protein. After 30 min at 37°C, the cells were washed three times in complete medium and analyzed by flow cytometry.

### Viral infections

5×10^5^ MDdMφ and MDDC were exposed to DV serotype 1 (strain FGA/NA d1d) [11], serotype 2 (strain 16681), or serotype 3 (strain PaH 881, isolated in 1988 in Thailand) in RPMI medium supplemented with 0.2% bovine serum albumin for 2 h. Viral growth was determined at 40 h post-infection. Virus titration was performed as previously described [3]. Infectivity titers were expressed as focus forming unit (FFU) on mosquito AP61 cell line (DV1 and DV3) or plaque forming unit (PFU) on mammalian BHK cell line (DV2). Different titering assays were performed to independently confirm our findings, despite the fact both methods may not be equivalent. The limit of titer determination was fixed at 10^3^, below which viral production was considered non-significant. For FACS analysis, infected cells were fixed and labeled for intracellular viral antigens with antiserum raised in mice that had received intracerebral DV injection [3]. IFN-α released from DV1-infected MDdMφ and MDDC was measured by ELISA (R&D Systems).

### Microscopy on cultured cells

To observe live DV internalization by MDDC and MDdMφ, the cells were exposed to DV1 at an MOI of 100 at 4°C for 30 min or at 37°C for 1 h and fixed in 2.5% glutaraldehyde. Cells were postfixed in osmium tetroxide, dehydrated in ethanol containing 1% uranyl acetate, treated with propylene oxide and embedded in resin (Durcupan ACM, Fluka). Ultrathin sections were stained with lead citrate and examined by transmission electron microscopy (TEM) (Hitachi H600). Images were acquired using a CCD camera (Hamamatsu). To visualize DV3 sE-eGFP internalization and endosomal acidification, cells were incubated with 10 µM LysoSensor Blue DND-167 (Molecular Probes-Invitrogen) for 30 min at 37°C. Protein sE-eGFP was added at a concentration of 3 µg/ml, and cells were viewed after different incubation times using a confocal microscope (LSM510, Zeiss). The blue color emitted by the LysoSensor dye was digitally converted into red. For TEM, cells were fixed in 2% paraformaldehyde and 0.2% glutaraldehyde. Cells were embedded in 1% agarose, permeabilized with 0.2% saponin and saturated with 2% BSA before incubation with 5 µg/ml polyclonal rabbit anti-GFP antibody (Rockland). The antibody was visualized by pre-embedding labeling using a goat anti-rabbit IgG conjugated to 0.8 nm gold particles, according to manufacturer's instructions (Aurion). Cells were fixed in 1% glutaraldehyde, and gold particles were enhanced using a silver kit (HQ silver, Nanoprobes). Cells were then treated and observed as above.

## Results

**Figure 1:**
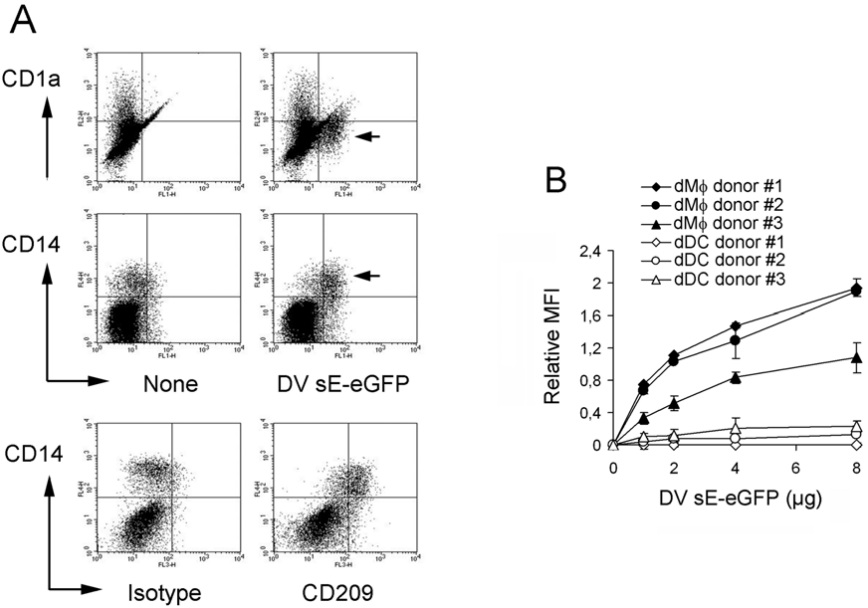
Dermal Mφ bind DV3 sE protein. A. The single-cell dermal suspension was incubated without (None) or with 2 µg of DV3 sE-eGFP fusion protein for 30 min at 37°C, washed, labeled for CD1a and CD14, and analyzed by flow cytometry. Live and large cells were pre-gated using the FSC/SSC channels. To test for CD209 expression, the cell suspension was incubated with anti-CD209 mAb or its isotype control together with anti-CD14 mAb and analyzed by flow cytometry. Arrows point to DV3 sE protein^+^ cells. The data is representative of 4 donors. B. The cell suspension was incubated with increasing amounts of DV3 sE-eGFP protein before labeling for CD1a, CD14 and HLA-DR. The relative mean fluorescence intensity (MFI) of DV3 sE protein was determined for CD14^+^HLA-DR^+^ (dMφ) and CD1a^+^HLA-DR^+^ (dDC) cells and shown as a function of DV3 sE-eGFP protein concentration. The analysis was performed in triplicate (mean±SD) for 3 donors.

We wished to determine whether human dMφ are targets of DV infection. To this end, healthy human skin from patients undergoing plastic surgery was processed to obtain a dermal cell suspension. The cells were then cultured without additional cytokines for 48 h to allow re-expression of cell surface markers, such as CD1a and CD209, lost during the collagenase treatment (data not shown). Binding of DV3 E protein to dermal cells was assessed by flow cytometry after staining with CD14 and CD1a-specific antibodies. CD14 is expressed by dMφ and CD1a by dDC [7]–[9]. To detect E protein binding, the soluble form of DV3 E protein (sE) was fused to the reporter protein eGFP and purified from a *Drosophila* expression system. As shown in **Figure 1A**, CD1a^+^ dDC showed only a limited capacity to interact with DV3 sE protein, whereas CD14^+^ dMφ readily bound the protein. This is corroborated by the distinct expression of CD209 by dMφ (**Fig. 1A**), whereas dDC expressed little, if any, CD209 (data not shown). Increasing amounts of DV3 sE protein were added to the dermal cell suspension to test if dDC bound the protein at higher concentrations. **Figure 1B** shows that even at high concentrations, there was little binding of DV3 sE protein to dDC, whereas it bound to dMφ in a dose-dependent fashion. These findings identify dMφ as potential key cellular targets of DV.

**Figure 2:**
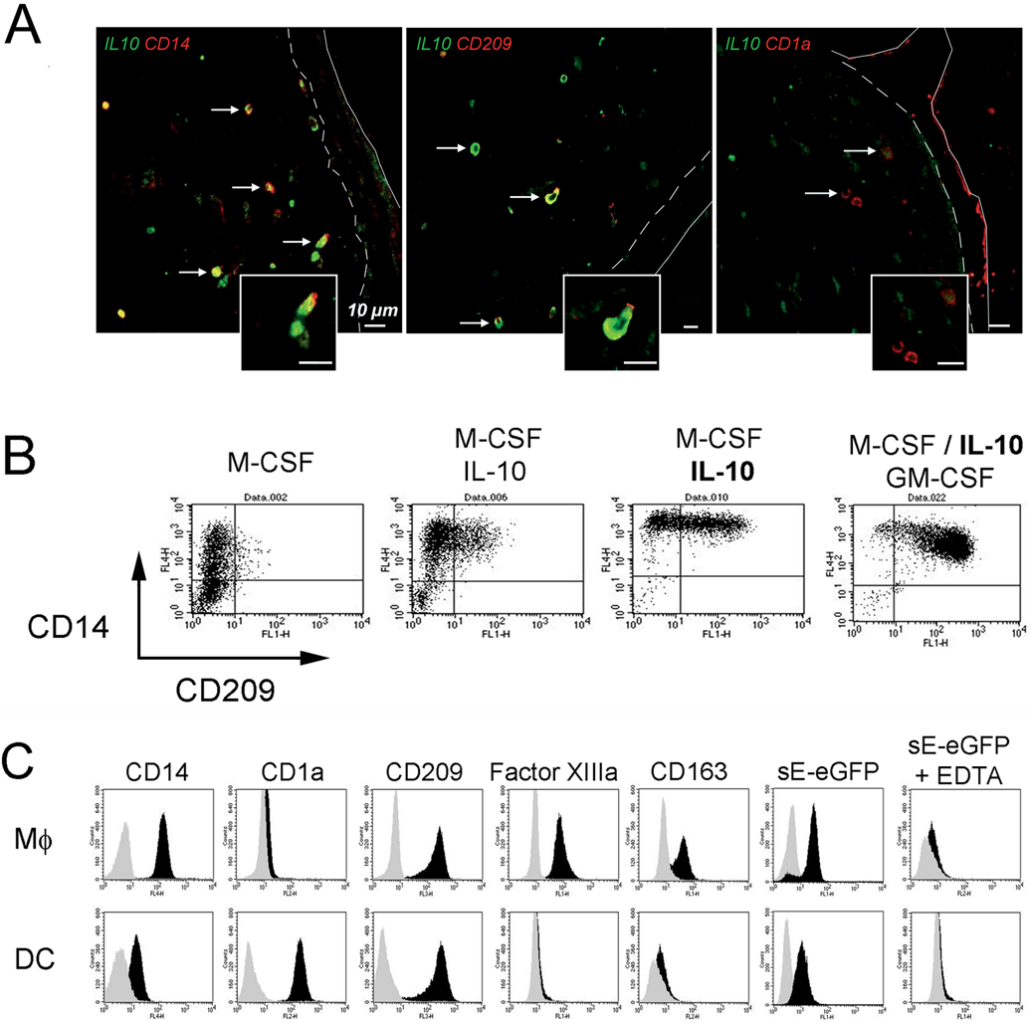
Generation of dermal-type Mφ with IL-10. A. Human CD14^+^ and CD209^+^ dMφ stained for IL-10. Formaldehyde-fixed sections of human skin were incubated with anti-IL-10 goat Ab and anti-CD14, anti-CD209 or anti-CD1a mAb. The goat Ab was detected using a biotin-labeled donkey anti-goat Ab, followed by streptavidin-Alexa 488, and the mAbs were revealed by rabbit anti-mouse Ab followed by Cy3-donkey anti-rabbit Ab. The data is representative of 3 donors. The dotted line delimits the dermo-epidermal junction. B. Adult blood monocytes were cultured for 5 days in medium containing fetal calf serum and 10 ng/ml M-CSF without or with a low (10 ng/ml) or a high (30 ng/ml, bold type) concentration of IL-10 in the absence or presence of 30 ng/ml GM-CSF. Non-adherent cells were collected and, after gating for viable cells, were analyzed for expression of CD14 and CD209. Dot plot quadrants were placed according to isotype controls. C. Dermal-type Mφ were generated in M-CSF/IL-10/GM-CSF as in panel B and compared to DC obtained from monocytes in GM-CSF and IL-4. Expression of cell surface markers was measured by FACS. Specific staining is in black and isotype controls are in grey. DV3 sE-eGFP protein binding was measured by FACS after incubating the cells with DV3 sE protein in the absence or presence of EDTA. Shown in black is the fluorescence of cells incubated with DV3 sE-eGFP protein and in grey is fluorescence of cells without the fusion protein.

To address the question of whether dMφ are infected by DV and whether they are permissive for viral production, we established cell culture conditions to generate dermal-type Mφ from monocytes. We observed on human skin tissue sections that dMφ expressing CD14 or CD209, but not the CD1a^+^ dDC, stained for IL-10 (**Fig. 2A**). When purified human monocytes were cultured in M-CSF and increasing concentrations of IL-10, the cells expressed CD14 and CD209 in an IL-10 dose-dependent manner (**Fig. 2B**). Similar to DC [12], the addition of GM-CSF increased CD209 levels (**Fig. 2B**), so that a homogeneous CD14^+^CD209^+^ cell population could be obtained with CD209 expression nearly identical to that of DC derived from monocytes in the presence of GM-CSF and IL-4 (**Figure S1A**). Western blotting of cell lysates confirmed the presence of CD209 as a major band of 49 kDa in both cell-types [13] (**Figure S1B**). The Mφ expressed coagulation factor XIIIa and CD163, two other cell surface markers of dMφ [14] (**Fig. 2C**). The Mφ and the DC were both able to bind eGFP-tagged DV3 sE protein, which was inhibited by EDTA (**Fig. 2C**). This distinguishes the monocyte-derived DC from dDC. Upon activation by lipopolysaccharide (LPS), the Mφ rapidly released IL-10, whereas DC or monocytes produced little of this cytokine (**Figure S1C**).

**Figure 3:**
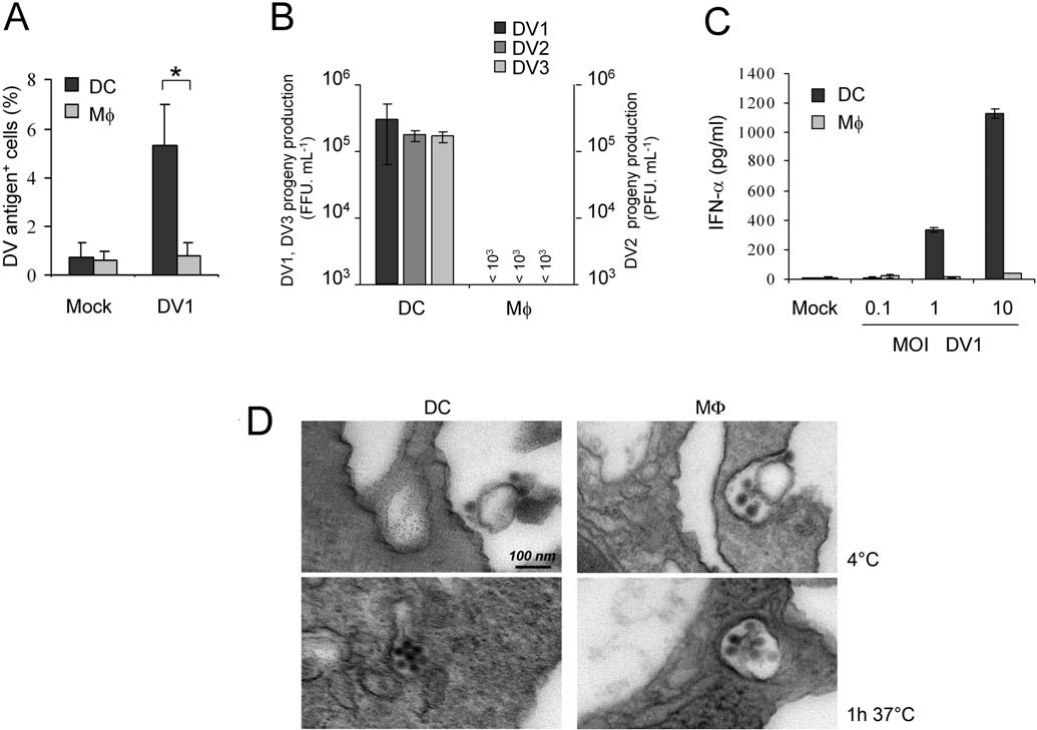
MDdMφ are resistant to permissive DV infection. A. 5×10^5^ MDDC and MDdMφ were exposed to DV1 at an MOI of 1 for 2 h, washed, and after 40 h processed for intracellular DV antigen detection by FACS. Graph shows the mean %±SD of intracellular DV antigens from 4 donors. * is significant (p<0.05) by the two-tailed student's *t*-test. B. Focus/plaque-forming assays (FFU/PFU) showing the mean titers±SD of DV1, 2 and 3 from supernatants of MDDC and MDdMφ (3 donors each). 5×10^5^ cells were exposed to DV1 and DV3 at an MOI of 1 and to DV2 at an MOI of 2 for 2 h, washed, and after 40 h, the supernatant was collected. The infectious titer was determined on AP61 cells (DV1 and DV3) and on BHK cells (DV2). C. IFN-α secretion of non-infected (Mock) or infected MDDC and MDdMφ was determined with increasing MOI of DV1. The data is expressed as the mean±SD of triplicate values and is representative of 3 donors. D. Both cell-types were exposed to DV1 at an MOI of 100. Transmission electron micrograph showing virions bound to plasma membranes at 4°C, and internalization into vesicles 1 h after exposure to virus at 37°C.

Monocyte-derived dMφ (MDdMφ) and monocyte-derived DC (MDDC) were analyzed for DV infection using low-passage DV1 and DV3 strains grown in mosquito cells [3] as well as the prototype DV2 strain 16681 [15]. The cells were exposed to DV1 at a multiplicity of infection (MOI) of 1 for 2 h, washed, and then cultured for 40 h. As shown in **Figure 3A**, intracellular viral antigen was clearly detected in MDDC by flow cytometry, whereas no specific immuno-labeling was observed in MDdMφ. An analysis of DV replication in these cells infected at an MOI of 1 (DV1 and DV3) or 2 (DV2) showed that MDDC were highly permissive to productive infection (∼10^5^ FFU/ml or PFU/ml) (**Fig. 3B**); in contrast, progeny virus production was undetectable in DV-infected MDdMφ (<10^3^ FFU/ml or PFU/ml). Consistent with this finding, no IFN-α was produced by DV-infected MDdMφ, even at an MOI of 10, whereas MDDC readily released IFN-α when infected with DV at an MOI of 1 or 10 [16] (**Fig. 3C**). To verify that MDdMφ acquired the virus, both myeloid cell-types were exposed to high DV input (MOI of 100) and electron microscopy analysis was performed after 30 min at 4°C and after 1 h at 37°C (**Fig. 3D**). Cell surface-bound (at 4°C) and endosomal vesicle-associated virus particles (at 37°C) were clearly detected in both cell-types. Thus, internalization of DV can occur in MDdMφ but does not result in productive infection.

**Figure 4:**
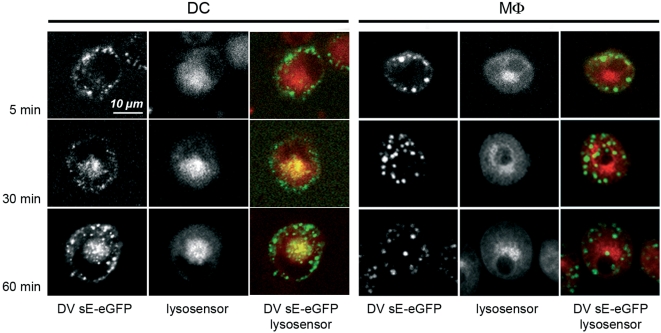
DV3 sE-eGFP protein is excluded from acidic compartments in MDdMφ. Confocal microscopic analysis of the localization of DV3 sE-eGFP protein at different time-points in cells loaded with the fluorescent LysoSensor dye (optimal fluorescence at pH 5.1). For the overlay images, blue color was converted to red to allow better co-visualization with eGFP. After 5 min incubation, sE-eGFP was endocytosed into vesicles close to the plasma membrane in both cells types. After 30 min and 60 min, sE-eGFP-containing vesicles acidified in the perinuclear area in MDDC, whereas in MDdMφ, sE-eGFP remained in non-acidified, large vesicles. The data is representative of 3 experiments.

**Figure 5:**
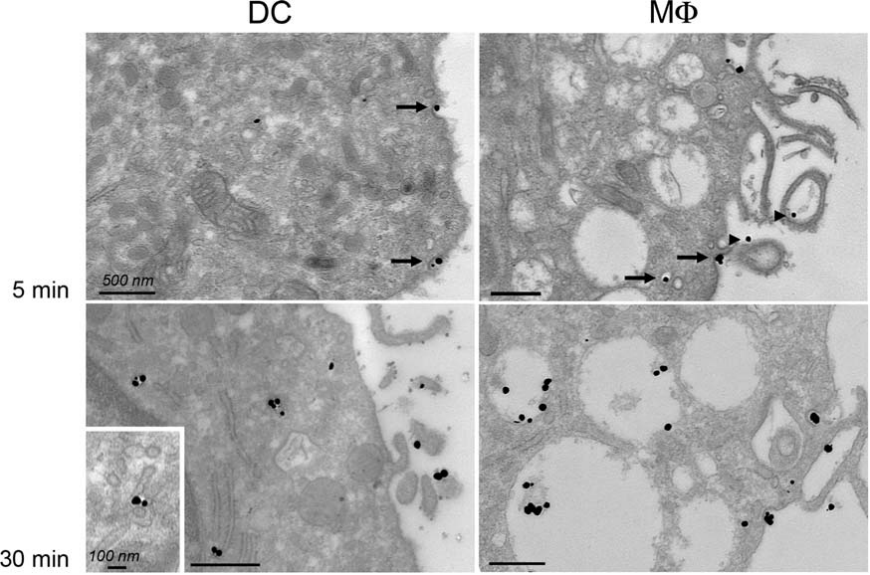
DV3 sE-eGFP protein-containing vesicles are distinct in MDDC and MDdMφ. Cells were processed for electron-microscopic localization of sE-eGFP using gold-conjugated anti-GFP Ab followed by silver enhancement. Five min after incubation with sE-eGFP, the protein was bound to the plasma membrane (arrow heads) and entered both cell-types in small endosomes, invaginated from the plasma membrane (arrows). At 30 min, in MDDC, sE-eGFP was localized to small, peri-nuclear endosomes, often associated with the ER, whereas in MDdMφ, the sE-eGFP was concentrated in large phagosomes. The inset shows a high-magnification view of sE-eGFP inside a tubular-shaped vesicle.

In an effort to define the molecular basis of the inability of DV to grow in MDdMφ. we asked whether internalized DV was sequestered in a manner that hampers productive infection, using DV3 sE-eGFP fusion protein. To monitor DV3 sE protein internalization in MDdMφ and MDDC, the cells were incubated with pH-sensitive LysoSensor dye and analyzed by confocal microscopy (**Fig. 4**). This dye accumulates in acidic organelles, where its fluorescence emission is highest. After 5 min at 37°C, DV3 sE protein was observed in vesicle-like structures in both cell-types. By 30 min and 60 min, DV3 sE protein dispersed to acidified perinuclear lysosomes in MDDC. In marked contrast, when MDdMφ were examined at these time-points, a large fraction of internalized DV3 sE protein was excluded from the acidic compartment and remained in non-acidic, large endosomes. Electron microscopy analysis using a colloidal gold-conjugated antibody to GFP demonstrated that DV3 sE protein accumulated in large phagosomes in MDdMφ, located close to the plasma membrane (**Fig. 5**). On the other hand, at 30 min, in MDDC, DV3 sE protein was mostly found in small perinuclear vesicles in the environment of the endoplasmic reticulum. Taken together, these data suggest that the inability of DV to productively infect MDdMφ is due to accumulation of virus particles in immature endosomal vesicles whose pH does not allow efficient viral-cell membrane fusion and subsequent virus uncoating.

## Discussion

In the present study, we demonstrated for the first time the interaction of dMφ with DV3 sE glycoprotein, which correlates with the expression of the DV attachment receptor CD209. Dermal DC displayed only a limited capacity to interact with DV3 sE protein and expressed little CD209. In accordance with these findings, *in situ* immuno-labeling of human skin section revealed CD209 expression by dMφ but little on DC [7]–[9]. Both cell types carry the MR [7], which also recognizes DV E protein [5]. Due to the nature of our binding assay, the dermal cells with the highest affinity for DV3 sE protein would acquire the most DV3 sE protein, suggesting that dDC may capture the recombinant envelope protein when physically isolated from dMφ. In the skin, the abundance, the location and the co-expression of CD209, L-SIGN and MR are likely to determine the nature of the DV-capturing immune cell.

Based on the observations that dMφ stained for intracellular IL-10 *in situ* and that IL-10 is produced by dMφ *ex vivo* [17],[18], we tested the effect of IL-10 on the formation of dMφ from monocytes. By combining IL-10, M-CSF and GM-CSF, a homogenous cell population was obtained which carried CD209 and other markers characteristic of dMφ, rapidly produced IL-10 in response to LPS or other toll-like receptor ligands (data not shown), and bound DV3 sE protein. Like MDDC, the MDdMφ were capable of internalizing live DV but, distinct from MDDC, they displayed an inherent resistance to viral growth. In contrast to DV3 sE protein found in acidified compartments in MDDC, we observed that DV3 sE protein accumulated in non-acidified phagosomes in MDdMφ. The DC vesicles containing DV3 sE protein or live virus were bell-shaped or tubular, whereas they were round, larger and close to the plasma membrane in the Mφ. To our knowledge, this identifies MDdMφ as the first innate immune cell capable of protecting the human host from DV infection and virus propagation. From this data, we propose that dMφ can act to trap infecting virions in a fusion-incompetent endosomal environment and thus to prevent DV spread to dDC at the anatomical site of the mosquito bite. We cannot formally exclude the possibility that downstream delays in the viral life cycle contribute to the inability of DV to replicate in MDdMφ, but the finding that West Nile virus productively infects these cells (data not shown) indicates that they are not generally refractory to flavivirus growth.

IL-10, required for CD209 expression and blockage of endosome acidification, is likely to be produced by the dMφ themselves, constitutively, or in response to stimuli such as UV-light [17]. In this context, a key question is whether mosquito salivary proteins, co-injected with the infectious virus, would also trigger IL-10 production by dMφ or, on the contrary, provoke an inflammatory response. Inflammatory cytokines of the Th2 T-helper cell type, IL-4 and IL-13, may be responsible for the formation of CD209^+^MR^+^ DC, which are permissive for DV infection and viral progeny production [3]–[6]. Alternatively, the presence of anti-DV non-neutralizing antibodies raised against a heterotypic DV serotype may render dDC susceptible to DV infection at the site of the mosquito bite.

The abundance and strategic position of the Mφ in the dermis is consistent with their function as first defense barrier against pathogens by isolating and eliminating them and thus avoiding unnecessary immune activation. However, other pathogens that recognize C-type lectins, such as mycobacteria, may exploit these cells to escape immune attack. Accumulating CD209^+^ Mφ in leprosy skin lesions have been associated with mycobacterial persistence [19]. Important questions to address in future are whether DV is eliminated in MDdMφ, whether infected MDdMφ gradually release DV, as shown for the foot-and-mouth disease virus and pulmonary Mφ [20], and whether rapid DV growth can occur when the Mα convert to DC. Improved knowledge of the molecular mechanisms for suppressing pathogen growth in MDdMφ will provide new insight into the crucial role of dMφ in protective immunity to infectious agents at the skin level.

## Supporting Information

Figure S1 Comparison of monocyte-derived DC and dermal-type macrophages
(0.95 MB TIF)

Alternative Language Abstract S1 Translation of the abstract into French by M. Decossas
(0.02 MB DOC)

Alternative Language Abstract S2 Translation of the abstract into Spanish by E. Navarro-Sanchez
(0.02 MB DOC)

Alternative Language Abstract S3 Translation of the abstract into German by C. Mueller
(0.02 MB DOC)
