# Supplementary figures and images for: Dermal-Type Macrophages Expressing CD209/DC-SIGN Show Inherent Resistance to Dengue Virus Growth
Source: PLoS Negl Trop Dis. 2008 Oct 1;2(10):e311. doi: 10.1371/journal.pntd.0000311 (PMC2553280; doi:10.1371/journal.pntd.0000311)

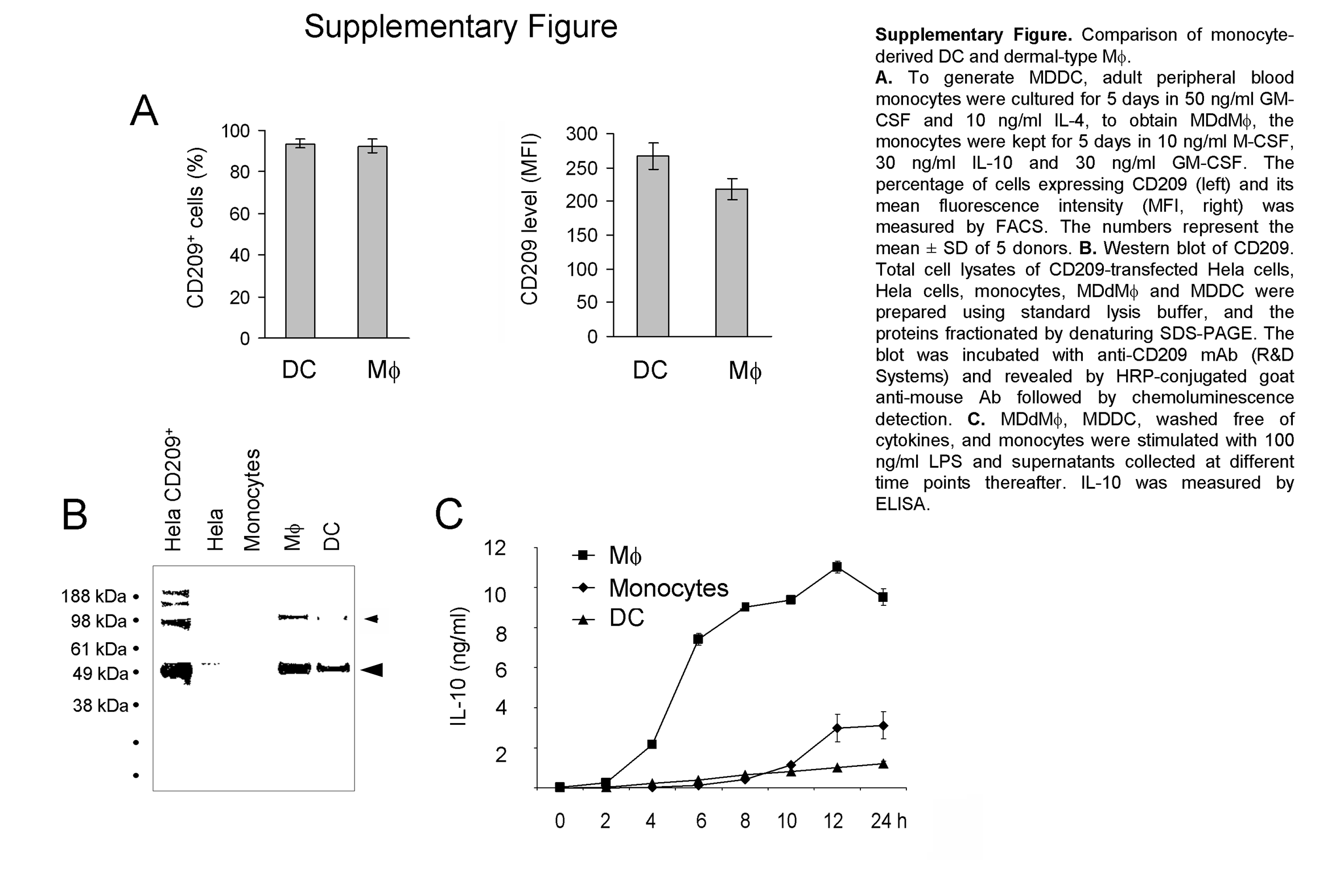

Supplement: Figure S1 — Comparison of monocyte-derived DC and dermal-type macrophages (0.95 MB TIF) [file pntd.0000311.s001.tif]
