# Supplementary material for: Dermal-Type Macrophages Expressing CD209/DC-SIGN Show Inherent Resistance to Dengue Virus Growth
Source: PLoS Negl Trop Dis. 2008 Oct 1;2(10):e311. doi: 10.1371/journal.pntd.0000311 (PMC2553280; doi:10.1371/journal.pntd.0000311)
Supplement: Alternative Language Abstract S1 — Translation of the abstract into French by M. Decossas (0.02 MB DOC) [file pntd.0000311.s002.doc]

Une question importante pour la compréhension de la pathogenèse de la Dengue concerne l’identité des cellules immunes impliquées dans le contrôle de l’infection par ce virus au site de la piqûre du moustique. Il semble que l’infection des cellules dendritiques immatures joue un rôle crucial dans la pathogenèse de la Dengue et que l’interaction entre la glycoprotéine E de l’enveloppe virale et CD209/DC-SIGN soit un élément clé pour leur infection productive. Bien que les macrophages dermiques expriment CD209, peu de données concernent leur rôle dans l’infection par la Dengue. Dans ce travail nous avons montré que les macrophages dermiques fixent la glycoprotéine E recombinante fusionnée à la GFP. Etant donné que les macrophages dermiques sont marqués pour l’IL-10 *in situ*, nous avons généré des macrophages de ce type à partir de monocytes en présence d’IL-10 et avons étudié leur infection par le virus de la Dengue. Ces macrophages sont capables d’internaliser le virus, mais aucune production virale n’est détectée dans les cellules infectées. De plus, l’INF-α n’est pas produit en réponse au virus. L’incapacité du virus à se reproduire dans les cellules a pu être attribuée à l’accumulation du virus internalisé dans des phagosomes peu acides. Un tel mécanisme présenterait un moyen nouveau de contenir l’infection des virus enveloppés et pourrait constituer un système de défense important pour empêcher la dissémination du virus de la Dengue juste après la piqûre du moustique.
