# Supplementary material for: Dermal-Type Macrophages Expressing CD209/DC-SIGN Show Inherent Resistance to Dengue Virus Growth
Source: PLoS Negl Trop Dis. 2008 Oct 1;2(10):e311. doi: 10.1371/journal.pntd.0000311 (PMC2553280; doi:10.1371/journal.pntd.0000311)
Supplement: Alternative Language Abstract S2 — Translation of the abstract into Spanish by E. Navarro-Sanchez (0.02 MB DOC) [file pntd.0000311.s003.doc]

Una importante pregunta en la patogénesis de la enfermedad del dengue es la identidad de las células inmunes implicadas en el control de la infección por el virus del dengue en el sitio anatómico de la picadura del mosquito. Existe la evidencia que la infección de las células dendríticas mieloides inmaduras juegan un papel crucial en la patogénesis de la enfermedad del dengue y la unión de la glicoproteína E de la envoltura viral con CD209/DC-SIGN es un elemento clave para su infección productiva. Los macrófagos de la dermis expresan CD209 sin embargo su papel durante la infección por el virus del dengue es poco conocido. Nosotros mostramos que los macrófagos de la dermis interaccionan con la glicoproteína recombinante E viral fusionada a la proteína verde fluorescente. Por que *en situ*, los macrófagos de la dermis son positivos a la tinción de IL10, nosotros generamos macrófagos dermales a partir de monocitos en presencia de IL10 y estudiamos su infección por el virus del dengue. Los macrófagos fueron capaces de internalizar el virus, pero no se detecto una producción de la progiene viral en las células infectadas. Además no se produjo IFN**-**α en respuesta a la infección. La incapacidad del virus del dengue de infectar los macrófagos fue debida a la acumulación de las partículas virales internalizadas en los fagosomas pobremente acidificados. Tal mecanismo presenta un nuevo significado en la infección de los virus envueltos y puede constituir un sistema de defensa primario para prevenir la extensión del virus del dengue poco después de la picadura por un mosquito infectado.
