# Supplementary material for: Dermal-Type Macrophages Expressing CD209/DC-SIGN Show Inherent Resistance to Dengue Virus Growth
Source: PLoS Negl Trop Dis. 2008 Oct 1;2(10):e311. doi: 10.1371/journal.pntd.0000311 (PMC2553280; doi:10.1371/journal.pntd.0000311)
Supplement: Alternative Language Abstract S3 — Translation of the abstract into German by C. Mueller (0.02 MB DOC) [file pntd.0000311.s004.doc]

Eine wichtige Frage für das bessere Verständnis der Pathogenese des Dengue Virus ist die Identität der Immunzellen, die an der Kontrolle der Virusinfektion an der Stelle des Moskitostiches beteiligt sind. Es gibt Anhaltspunkte, die zeigen, dass unreife dendritische Zellen eine ausschlaggebende Rolle bei der Dengue Virus Infektion spielen and dass die Wechselwirkung zwischen dem viralen Membranprotein E und CD209/DC-SIGN ein Schlüsselelement für die produktive Infektion sind. Obwohl dermale Macrophagen CD209 exprimieren, ist über deren Rolle bei Dengue Virus Infektionen wenig bekannt.

Wir habe hier gezeigt, dass dermale Macrophagen das Membranprotein E binden, das mit dem grün fluoreszierendem Protein fusioniert ist. An Hand der Beobachtung, dass dermale Macrophagen sich für IL-10 *in situ* färben lassen, haben wir dermale Macrophagen aus Monozyten gezogen und haben deren Infektion mit Dengue Viren untersucht. Die Macrophagen konnten zwar die Viren aufnehmen aber eine Virusproduktion war für diese infizierten Zellen nicht nachweisbar. Außerdem konnte kein IFN-α nachgewiesen werden. Die Unfähigkeit des Virus sich in den Macrophagen zu vermehren konnte darauf zurückgeführt werden, dass das Virus sich in nur schwach sauere Phagosomen ansammelt. Ein derartiger Mechanismus wäre ein neues Mittel die Infektion von membranen Viren zu beschränken und könnte eine wichtige Abwehr bilden um die Verbreitung des Dengue Virus kurz nach dem Moskitostich vorzubeugen.
